# Physical Activity, Sport Practice and Cardiovascular Risk Factors in Workers From a Public Hospital: A Trajectory Analysis

**DOI:** 10.3389/fcvm.2021.740442

**Published:** 2021-12-17

**Authors:** Rui Gonçalves Marques Elias, Ana Silvia Degasperi Ieker, Lucas Lopes dos Reis, Antônio Stabelini Neto, Jeffer Eidi Sasaki, Wendell Arthur Lopes, Carla Eloise Costa, Wilson Rinaldi

**Affiliations:** ^1^UENP Laboratory - State University of Northern Paraná, Department of Health Sciences, Organization Research Group on Lifestyle, Exercise and Health - GPVES/UENP, Jacarezinho, Brazil; ^2^Master's Program in Physical Education, State University of Maringá, Maringá, Brazil; ^3^Laboratory UNIPAR - Paraná University, Strategy for the Promotion of Health, Strategy for the Promotion of Health, Coexistence With Diversity in the University Community (SACODI), Umuarama, Brazil; ^4^Student of the Postgraduate Program in Physical Education, State University of Maringá and State University of Londrina, Maringá, Brazil; ^5^Laboratory UFTM, Federal University of Triangulo Mineiro, Uberaba, Brazil; ^6^Laboratory of State University of Maringá, Department of Human Movement Sciences, Regional Campus of Vale do Ivaí, Member of the Research Group on Systemic Arterial Hypertension, Arterial Stiffness and Vascular Aging (GPHARV), Maringá, Brazil; ^7^Laboratory of State University of Maringá, Research Group Laboratory of Studies in Physical Exercise and Health DEF / UEM / National Council for Scientific and Technological Development (CNPq), Maringá, Brazil

**Keywords:** longitudinal studies, occupational health, chronic disease, health promotion, cardiovascular risk

## Abstract

**Background:** Studies have demonstrated the positive effects of physical activity on cardiovascular risk factors. Longitudinal studies using modeled trajectories are necessary to understand patterns of physical activity and association with cardiovascular risk factors.

**Objective:** To analyze the association between sports practice in young people and current physical activity with the trajectory of cardiovascular risk factors in workers at a public hospital.

**Methods:** Four hundred and seventeen workers was followed for four years reporting Physical Activity, health status, lifestyle behaviors and socio-demographic characteristics. Group-based trajectory modeling identified the trajectories of PA and associations with time-stable and time-varying covariates. We considered a range of sociodemographic and health and lifestyle factors as potential covariates.

**Results:** The results shows the association between participation in sports activities in youth and current physical activity and trajectories of cardiovascular risk, adjusted for sex and age (*p* < 0.05). Adults who reported having played sports in their youth and are currently active have a lower risk of having a history of obesity and low HDL-c than workers who did not play sports in their youth and are currently sedentary 0.690 (0.565–0.844) obesity, 0.647 (0.500–0.837) low HDL-c.

**Conclusion:** The practice of sports in youth and current physical activity is a protective factor against the trajectory of obesity and low HDL-c, mainly in female workers. Programs to encourage the practice of physical activity should be carried out in order to reduce cardiovascular risk factors and prevent chronic diseases in workers.

## Introduction

Chronic non-communicable diseases (NCDs) are the main causes of death worldwide, and among them, cardiovascular disease is among the most prevalent (1). In Brazil, NCDs are also a public health problem, being responsible for 72% of deaths, most of which are caused by diseases of the circulatory system and neoplasms (2, 3).

Among the determinants for NCD diseases, physical inactivity has received special attention. Data from the World Health Organization (1), point out that 23% of adults are insufficiently active. Insufficient physical activity contributes to 3.2 million deaths and 69.3 million years of life lost due to disability (DALYs). Physical inactivity has also been researched in the occupational setting. Physically inactive individuals with excess weight can reduce the risk of disability at work by 20% when they become active (4).

Experiences in different types of activities, such as active transport or sports, can increase adherence and maintenance of physical activity. In addition, the literature describes a possible direct effect of sports practice in childhood on chronic diseases during later ages (5). Studies have demonstrated the cumulative effects of physical activity on cardiovascular risk factors (6, 7). Existing studies on cardiovascular risk factors and physical activity normally measure prevalence or perform two assessments at the beginning and end, without intermediate measurements, in addition to the fact that most of them use clinical cutoff points to analyze data. The use of the trajectory model describes the behavior of the variable over the years. This could intensify prevention strategies against disease development (8). Some studys used group-based trajectory modeling (GBTM) to identify risk factors (9, 10). However, the longitudinal relationship between physical activity and cardiovascular risk factors is still unclear.

Longitudinal studies using modeled trajectories are needed to understand patterns of physical activity and association with cardiovascular risk factors. Therefore, the aim of this study was to analyze the association between sports practice in youth and current physical activity with the trajectory of cardiovascular risk factors in workers from a public hospital.

## Methods

### Study Participants

This was a retrospective cohort study of workers of public hospital at a teaching hospital in southern Brazil. The hospital has 797 permanent employees. This study was approved by the Human Research Ethics Committee under opinion number 1,766.685.

The study was separated into two moments: interview with the participants and analysis of the medical record. The inclusion criteria were: 1) workload of more than 30 h per week in the hospital and, 2) underwent medical consultation and delivering all periodic exams in three periods of time separated every two years. The exclusion criteria were employees that have undergone surgery, went through a period of pregnancy or was diagnosed with cancer.

After authorization from the hospital superintendent, the researchers received a list to employees, according to the work sector.

The researchers asked each person responsible for the board of directors the work schedule containing the name, sector, day and period of work. From then on, the study was advertised to the employees by e-mail and posters placed at the hospital.

According to work schedules, the researchers went to all sectors of work to conduct the interview, blood pressure assessment and anthropometric measurements with those volunteers who decided to take part in the study. When the employee was not in the sector for any reason, a new visit was scheduled, up to three attempts.

With this strategy, all employees were contacted about the survey by email, posters in the hospital and visit in the work sector. However, 58 employees did not participate in the survey; they were on leave, on vacation or relocated; 64 employees refused to participate and 43 were not found in the work sector in any of the three visits, totaling 485 interviews.

After the interviews, the 485 medical records of the Medical and Occupational Safety Service (SESMT) at the University were analyzed. Of these, 68 were incomplete and were excluded from the study.

**Figure 1:**
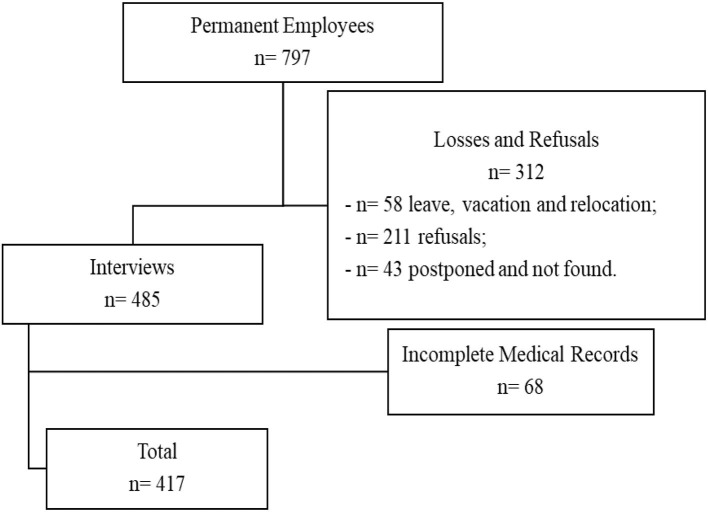
Diagram of representation of the population of servers.

Therefore, the final sample consisted of 417 employees interviewed who provided data from fully filled medical records (Figure 1).

### Study Variables

The data were collected by a team of five interviewers trained to apply the research instruments in a standard way. To this end, a 40-h training was conducted to standardize the application of questionnaires and anthropometric assessments.

Variables such as sex, age, work regime, working time, skin color and education were obtained by self-report by the participant. Through epidemiological studies, these independent variables sex, age, skin color, level of physical activity, were presented as high cardiovascular risk.

Current physical activity was assessed using the International Physical Activity Questionnaire (IPAQ-8 short version) in the form of an interview. The IPAQ-8 has been previously validated for application in Brazilian participants (11).

The current physical activity was classified into two categories (-0-) “Insufficiently Active,” those who do not met the minimum recommendations of 150 min/week of moderate to vigorous physical activity, and (-1-) “Active,” those who performed 150 min or more a week of moderate to vigorous physical activity (12). With the analysis of the short version of the IPAQ-8 questionnaire, it was possible to obtain the results of the participants' level of physical activity. Participants who reached ≥5 days of physical activity per week and added ≥150 min of practice during the week will be considered physically active, otherwise they were considered insufficiently active.

Previous sports practice (childhood and adolescence) was verified through with the following interview-based questions “did you systematically practice sports in childhood for at least one year?” “Did you systematically practice sports in your adolescence for at least one year?” Participants received the instruction to answer the questions considering the practice of a systematic sports outside the physical education classes at school. Participants should also report how many days a week and for how long they practiced, as well as the modality. Previous sports practice was classified into two categories (-0-) No, for those who answered both “no” for both questions and (-1-) Yes, for those who answered yes to at least one question.

For the analysis of previous sports practice and current physical activity, four groups were created: (0-0) Youth who did not practice sports and Adult Insufficiently Active; (1-0) Youth who practiced sports activities and Insufficiently Active Adult; (0-1) Young man who did not practice sports and Active Adult; and (1-1) Young man who did sports and Active Adult. Thus, the level of current physical activity was measured using the IPAQ-8 questionnaire and the level of previous physical activity was verified by questions about the sports activity that the participants performed in the past.

Body mass was assessed using a Welmy digital scale, to the nearest 100 grams, and height using a portable stadiometer, to the nearest 0.1 centimeters (13). To classify obesity, the Body Mass Index (BMI) measure above 30 kg/m^2^ was used.

Blood pressure values were taken after the individual was seated for more than 10 min. The measurements were taken twice and the lowest value was used. The automatic blood pressure monitor with digital reading of the OMRON model HEM-742INT was used. This instrument was validated by Coleman et al. (14), who reported a mean (standard deviation) difference between observer and device measurements of −1.15 ± 5.7 mmHg for systolic pressure and −1.61 ± 4.7 mmHg for diastolic pressure. The device was calibrated and compared with other devices once a week, as recommended by the manufacturer. The measurement procedures followed the recommendations of White et al. (15) and the American Heart Association (16).

Data on total cholesterol, HDL-c, LDL-c, triglycerides and glucose were collected from individual medical records, in three periods of time separated every two years. Thus, forming an analysis at year zero (y0), year two (t2) and year four (t4). To measure the dyslepidemic variable, a spectrophotometer machine was used. The cutoff point for dyslepidemic used to prevent atherosclerosis was: total cholesterol <190 mg/dl, HDL >40 mg/dl triglyceride mg/dl <150. In all analyses, the highest category was considered to be the civil servant who reported taking any antilipidemic or hypoglycemic medication.

The biochemical parameters were always evaluated in the same place, following the standard fasting methodology from 10 to 12 h, blood collected by venipuncture was distributed in anticoagulant tubes and stored for a maximum period of 48 h. The samples were centrifuged for evaluation by the colorimetric method.

### Data Analysis

Different statistical methods were applied and for all hypotheses tests we established the criterion of statistical significance at α = 5%. Descriptive statistics were displayed as mean values and 95% confidence intervals to characterize the population. The prevalence was calculated from the number of people with the outcome of interest divided by the total number of study participants.

Chi-square tests were used to analyze the association between the trajectory of cardiovascular risk (dependent) and sports practice in youth and current physical activity. The Chi-square of Mantel-Haenszel was used to verify the linearity of the variables and the Fisher's exact test was used when the sample size is small. The relative risk (RR) for the cardiovascular risk trajectory was obtained by the exponential of the regression coefficient and its 95% confidence interval performed using Poisson regression with robust variance, adjusted for sex and age.

Follow-up analysis (longitudinal trajectory of the variables): To characterize the longitudinal trajectory of the theoretical variables proposed in this study, an analysis of the group-based trajectory model (GBTM) was performed in the STATA TRAJ package to identify subgroups within each variable of interest (BMI), systolic and diastolic blood pressure, glucose, HDL-c, LDL-c total cholesterol and triglycerides. In the process of determining the number of groups, we initially used a quadratic model for all groups.

The final number of groups was determined based on the Bayesian Information Criterion (BIC), trajectory forms for similarity and the proportion of cohort members in each class (17). After identifying the ideal number of groups, the level of the polynomial for each group was reduced until a parameter estimate in the highest function had a *p*-value <0.01. With this final model, each participant was assigned to one of the subgroups based on the maximum subsequent probability. As a diagnosis of the model, four measures were used to assess the fit of the trajectory model, as suggested by Nagin (18): (i) the mean posterior probability of designation for each group is 0.7 or higher; (ii) the probabilities of correct classification are 5.0 or higher; (iii) the proportion of a sample attributed to a certain group is close to the proportion estimated from the model; and (iv) confidence intervals of 99% of the estimated proportion are reasonably narrow.

To estimate the association between exposure and outcome, we performed a double trajectory model that summarizes the dynamic interrelationship between two longitudinal variables in various trajectory groups, rather than a traditional association analysis that estimates the global association between two variables in heterogeneous subpopulations. All models included the converged double path model and all parameters had reasonably small standard errors (all standard errors divided by means were <0.3). Therefore, the initial values were not specified and the standard initial values were used.

## Results

Of the 417 workers interviewed, 65.50% (*n* = 273) were female. Regarding education, 69.9% had completed higher education. The average age was 48.43 ± 7.55 years and the average working time in the hospital was 18.67 ± 7.08 years. For the physical activity levels, 24.94% of the workers were considered physically active and 49.16% reported having practiced sports in their youth. Table 1 shows the characteristics of the subjects participating in the study.

**Table 1:** Characteristics of participating workers according to sex.

**Variables**	**Male** ***N* (%)**	**Female** ***N* (%)**	**Total** ***N* (%)**
**Age range**			
<40 years	29 (20.14)	36 (13.19)	65 (15.59)
41–50 years	64 (44.44)	134 (49.08)	198 (47.48)
>50 years	51 (35.42)	103 (37.73)	154 (36.93)
**Time at work**			
0–10 years	24 (16.67)	58 (21.25)	82 (19.66)
11–20 years	56 (38.89)	100 (36.63)	156 (37.41)
21–30 years	53 (36.81)	106 (38.83)	159 (38.13)
>30 years	11 (7.64)	09 (3.30)	20 (4.80)
**Skin color**			
White	106 (73.61)	209 (76.56)	315 (75.54)
Black	10 (6.94)	34 (12.45)	44 (10.55)
Another	28 (19.44)	30 (10.99)	58 (13.91)
**Schooling**			
Graduated	93 (64.58)	195 (71.43)	288 (69.06)
High school	33 (22.92)	68 (24.91)	101 (24.22)
Elementary school	18 (12.50)	10 (3.67)	28 (6.72)
**Work hours per day**			
6 h daily	56 (38.89)	136 (49.82)	192 (46.04)
8 h daily	47 (32.64)	45 (16.48)	92 (22.06)
12/36 h	41 (28.47)	92 (33.70)	133 (31.89)
**Sport and physical activity**			
0–0	53 (36.81)	111 (40.66)	164 (39.33)
1–0	53 (36.81)	96 (36.16)	149 (35.73)
0–1	13 (9.03)	35 (12.82)	48 (11.51)
1–1	25 (17.36)	31 (11.36)	56 (13.43)

*(0-0) Youth who did not engage in sports and an insufficiently active adult; (1-0) Youth who practiced sports activities and Insufficiently Active Adult; (0-1) Young man who did not practice sports and Active Adult and (1-1) Young man who did sports and Active Adult. %, Percentage*.

**Figure 2:**
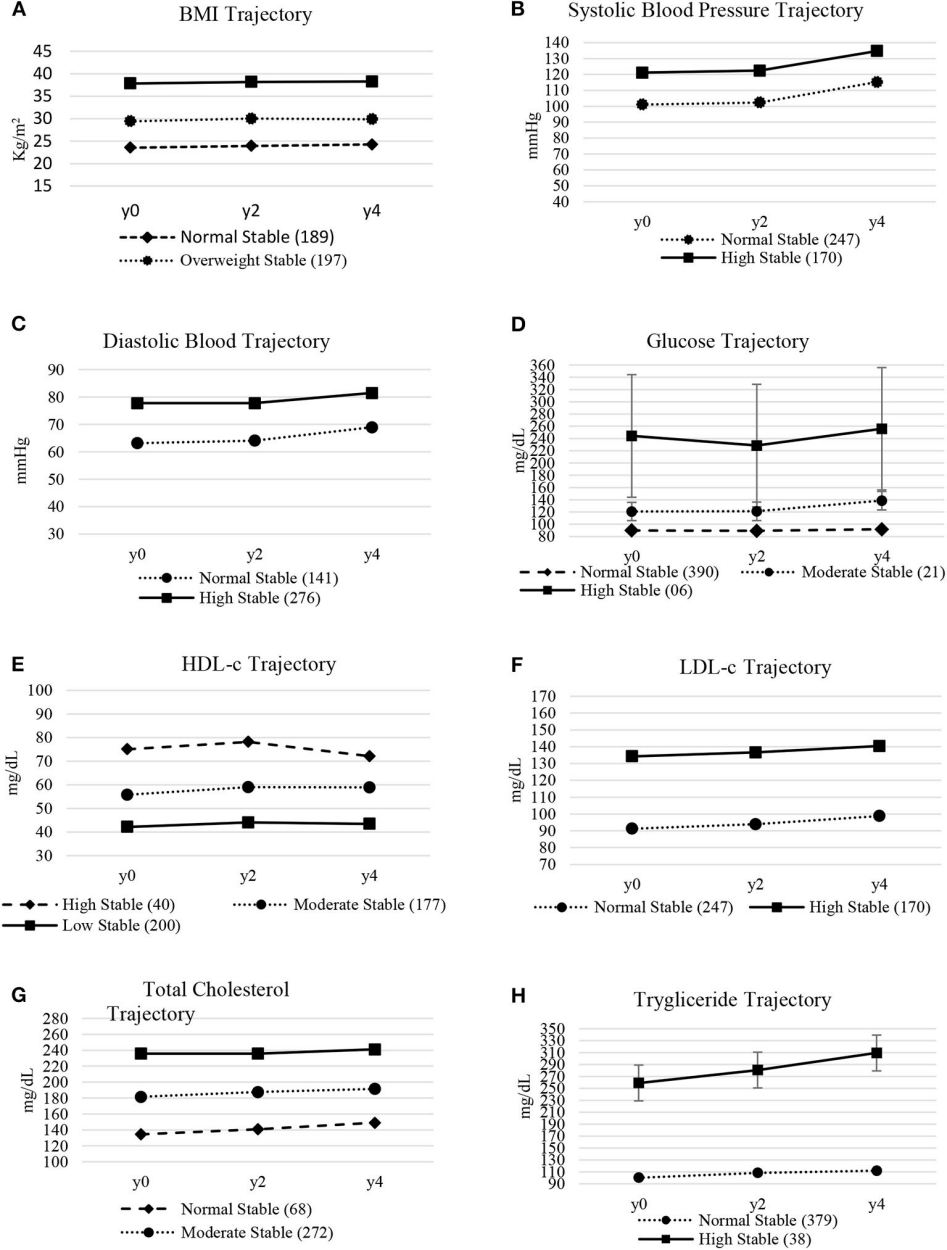
**(A–H)** Average and Confidence Interval (95% CI) of the trajectory of cardiovascular risk factors of workers in a public hospital.

To identify the trajectory model, the first step was to model the trajectory of the scores for cardiovascular risk factors. For each cardiovascular risk factor, two to three groups were created according to the longitudinal characteristics presented (Figure 2).

In the BMI, the analysis found three stable groups called “Normal Stable,” “Stable Overweight” and “Stable Obesity.” In the analysis of Glucose and Total Cholesterol, three stable groups were also found, named “Normal Stable,” “Moderate Stable” and “High Stable.” For HDL-c, the analysis found three stable groups named “High Stable,” “Moderate Stable” and “Low Stable.” In the analysis of Systolic Blood Pressure, Diastolic Blood Pressure, LDL-c and Triglyceride, two groups were found called “Normal Stable” and “High Stable.”

Table 2 shows the prevalence of cardiovascular risk trajectory according to participation in sports activities when young and current physical activity. No person who played sports in his youth and is currently active (1-1) has had a history of obesity and high glucose. Not having played sports in youth and currently being sedentary (0-0) had a higher prevalence in the trajectory of high diastolic blood pressure, high glucose level, low HDL-c, high LDL-c, high total cholesterol and high triglycerides. Being female was associated with the trajectory of Obesity, low HDL-c and high total cholesterol. The analysis, including female and male, showed an association in the trajectories of Obesity, low HDL-c and high total cholesterol, and also in high diastolic pressure.

**Table 2:** Trajectories of cardiovascular risk factors according to participation in sports activities in youth and current physical activity (%, 95% IC), separated according to sex.

**Male (*n* = 144)**	**0-0 (*n* = 53) % (IC 95%)**	**1-0 (*n* = 53) % (IC 95%)**	**0-1 (*n* = 13) % (IC 95%)**	**1-1 (*n* = 25) % (IC 95%)**	***P***
Obesity	5.66 (1.94–15.37)	9.43 (4.10–20.25)	0.00 (0.00–22.81)	0.00 (0.00–13.32)	0.442
High P.S.	56.60 (43.27–69.05)	62.26 (48.81–74.06)	38.46 (17.71–64.48)	64.00 (44.52–79.75)	0.416
High P.D.	81.13 (68.64–89.41)	88.68 (77.42–94.71)	69.23 (42.37–87.32)	68.00 (48.41–82.79)	0.120
High Glucose	3.77 (1.04–12.75)	2.04 (3.6–10.69)	0.00 (0.00–22.81)	0.00 (0.00–13.32)	0.647
HDL-c Low	73.58 (60.42–83.56)	60.38 (46.94–72.41)	76.92 (49.74–91.82)	52.00 (33.50–69.97)	0.151
High LDL-c	54.72 (41.45–67.34)	62.26 (48.81–74.06)	46.15 (23.21–70.86)	44.00 (26.67–62.93)	0.430
High CT	28.30 (17.97–41.57)	24.53 (14.93–37.57)	30.77 (12.68–57.63)	12.00 (4.17–29.96)	0.134
Trigli. High	16.98 (9.20–29.23)	18.87 (10.59–31.36)	7.69 (1.37–33.31)	8.00 (2.22–24.97)	0.523
**Female (*****n*** **=** **273)**	**0-0 (*****n*** **=** **111) % (IC 95%)**	**1-0 (*****n*** **=** **96) % (IC 95%)**	**0-1 (*****n*** **=** **35) % (IC 95%)**	**1-1 (*****n*** **=** **31) % (IC 95%)**	
Obesity	8.11 (4.32–14.69)	12.50 (7.30–20.59)	5.71 (1.58–18.61)	0.00 (0.00–10.72)	0.007^l*^
High P.S.	34.23 (26.07–43.46)	31.25 (22.85–41.09)	34.29 (20.83–50.85)	19.35 (9.19–36.28)	0.452
High P.D.	63.96 (54.70–72.29)	57.29 (47.30–66.72)	57.14 (40.86–72.02)	46.16 (29.16–62.23)	0.293
High Glucose	1.80 (0.50–6.33)	1.04 (0.18–5.67)	0.00 (0.00–9.89)	0.00 (0.00–11.03)	0.228
HDL-c Low	41.44 (32.71–50.74)	45.83 (36.22–55.77)	34.29 (20.83–50.85)	12.90 (5.13–28.85)	0.047^*a^
High LDL-c	63.96 (54.70–72.29)	29.17 (21.02–38.92)	60.00 (43.57–74.45)	29.03 (16.10–46.59)	0.557
High CT	19.82 (13.47–28.19)	13.54 (8.09–21.80)	17.14 (8.10–32.68)	3.23 (0.57–16.19)	0.012^a^
Trigli. High	8.11 (4.32–14.69)	5.21 (2.24–11.62)	2.86 (0.51–14.53)	3.23 (0.57–16.19)	0.562

(0–0) Youth who did not engage in sports and an insufficiently active adult; (1–0) Youth who practiced sports activities and Insufficiently Active Adult; (0–1) Youth who did not practice sports and Active Adult; (1–1) Youth who practiced sports and Active Adult; %, percentage; 95% CI, 95% Confidence Interval; High P.S., High Systolic Blood Pressure; High P.D., High Diastolic Blood Pressure; linear association; *association in the chi-square; High TC, High Total Cholesterol; Trigli. Elevated, Elevated Total Trilgiceride. The expression “a” significant linear association.

Table 3 shows the association between (1-1) participation in sports activities in youth and current physical activity and trajectories of cardiovascular risk, adjusted for sex and age. Adults who reported (1-1) having played sports in their youth and are currently active have a lower risk of having a history of obesity and low HDL-c, than workers (0-0) who did not play sports in their youth and are currently insufficiently active (reference group).

**Table 3:** Adjusted association between participation in sports activities in youth and current physical activity and the trajectory of cardiovascular risk factors of public workers.

**Male (*n* = 144)**	**0-0 (*n* = 53) % (IC 95%)**	**1-0 (*n* = 53) %(IC 95%)**	**0-1 (*n* = 13) %(IC 95%)**	**1-1 (*n* = 25) %(IC 95%)**
Obesity	Ref.	1.336 (0.845–1.723)	0.994 (0.708–1.394)	1.202 (0.938–1.541)
High SBP	Ref.	0.999 (0.798–1.250)	0.760 (0.549–1.054)	0.909 (0.726–1.139)
High DBP	Ref.	1.232 (0.924–1.507)	1.004 (0.734–1.373)	1.130 (0.911–1.404)
High glucose	Ref.	1.060 (0.946–1.188)	1.011 (0.856–1.195)	1.029 (0.911–1.162)
Low HDL-c	Ref.	1.128 (0.719–1.769)	1.446 (0.805–2.597)	1.423 (0.928–2.183)
High LDL-c	Ref.	1.235 (0.994–1.535)	0.985 (0.716–1.356)	1.080 (0.865–1.349)
High CT	Ref.	1.146 (0.904–1.453)	0.865 (0.524–1.427)	1.049 (0.818–1.345)
High TRI	Ref.	1.120 (0.969–1.295)	0.985 (0.822–1.181)	1.083 (0.935–1.255)
**Female (*****n*** **=** **273)**	**0-0 (n** **=** **111) % (IC 95%)**	**1-0 (*****n*** **=** **96) %(IC 95%)**	**0-1 (*****n*** **=** **35) %(IC 95%)**	**1-1 (*****n*** **=** **31) %(IC 95%)**
Obesity	Ref.	1.127 (0.910–1.397)	1.015 (0.844–1.220)	**0.690 (0.565–0.844)**
High P.S.	Ref.	1.023 (0.864–1.211)	1.054 (0.934–1.188)	0.973 (0.829–1.142)
High P.D.	Ref.	0.888 (0.676–1.168)	1.090 (0.950–1.250)	0.885 (0.713–1.098)
High glucose	Ref.	0.996 (0.891–1.114)	0.989 (0.906–1.080)	0.970 (0.889–1.060)
Low HDL-c	Ref.	0.883 (0.623–1.250)	1.115 (0.858–1.450)	**0.598 (0.436–0.821)**
High LDL-c	Ref.	1.066 (0.894–1.272)	1.012 (0.894–1.145)	1.036 (0.866–1.241)
High CT	Ref.	0.870 (0.692–1.094)	1.057 (0.910–1.228)	0.844 (0.729–1.071)
High TRI	Ref.	0.955 (0.888–1.027)	0.989 (0.926–1.056)	0.975 (0.900–1.055)

*(0-0) Youth who did not engage in sports and an insufficiently active adult; (1–0) Youth who practiced sports activities and Insufficiently Active Adult; (0–1) Youth who did not practice sports and Active Adult; (1–1) Youth who practiced sports and Active Adult; %, percentage; 95% CI, 95% Confidence Interval; High P.S., High Systolic Blood Pressure; High P.D., High Diastolic Blood Pressure; High TC, High Total Cholesterol; Trigli. Elevated, Elevated Total Trilgiceride; Ref., Reference category for association; A adjusted for sex and age. Female: adjusted only by age. In females for variable obesity, the classification (1–1) Young people who practice sports and Active Adult was associated with a value of 0.690 representing a cardiovascular risk protection value of 31%. Also in females, the Low HDL variable presented an association value of 0.598 representing a protection value of 40.2% for cardiovascular risk*.

## Discussion

The present study found that the trajectory of cardiovascular risk factors such as obesity and low HDL-c; are associated with current physical inactivity and/or not having practiced sports in youth.

Of the participants. 49.16% (*n* = 205) of the participants reported playing sports in their youth. however only 13.43% (*n* = 56) currently perform physical activity. A reduction of 72.27% (*n* = 149) of those who performed sports activities in youth. The literature has pointed out the importance of starting physical activity during childhood and carrying this behavior into adulthood and also at older ages (19). Adequate physical activity can prevent cardiovascular diseases and decrease the risk of mortality (5). The reduction in the practice of physical activity in adulthood may be associated with barriers related to time and place of residence (20, 21). In this specific study. The irregularity of hospital working shifts may be a justification for these findings. Therefore, the continuity of the practice of physical activity does not depend only on a personal effort the private sector and public sectors must facilitate this behavior.

Insufficient physical activity at all stages of life is a risk factor for the development of cardiovascular diseases. These risk factors must be analyzed based on longitudinal studies that use repeated measures, applied based on trajectories, which are able to follow the oscillations of the variables in different periods. Study by Dhana et al. (22) verified trajectories of increase and decrease in BMI. In the study by Allen et al. (9) the authors found four trajectories of systolic and diastolic blood pressure, which they defined as “High in Elevation,” “High Stable,” “Moderate Stable” and “Low Stable.”

In the study by Yuan et al. (23) in Chinese adults three categories were found. “Moderate glucose increase,” “strong increase and decrease” and “strong decrease and increase.” The trajectory models in the current study did not show changes in categories. The main characteristic being the stability of the cardiovascular risk factors evaluated. These differences may be related to the study segment time. Data from studies by Allen et al. (9), Dhana et al. (22) and Yuan et al. (23) were analyzed with an interval of five years. We also observe a stable behavior of the trajectories. However, it is possible to verify in the mentioned studies that, in the long run, even in models with moderate elevation are associated with cardiovascular diseases.

Observing the averages in the three moments of the trajectory of the present study, we can see a moderate increase in cardiovascular markers, which may indicate a significant increase in cardiovascular problems. The results justify the importance of investigations using the trajectory model to verify the behavior of risk factors over time.

The current study showed a significant association between the trajectory of obesity and low HDL-c. The group that had the lowest percentage in the trajectory of risk factors was (1-1), who reported having practiced sports when young and is currently active. Corroborating the findings by Nechuta et al. (19) who highlight the importance of sports practice in childhood/adolescence and continuity in adult life. Continuing to practice physical activity throughout life is a protective factor against cardiovascular disease.

Study by Elhakeem et al. (24) suggests that individuals who become active in adulthood improve the profile of cardiovascular markers in the elderly when compared to those who remain or become inactive. The changes include an improved lipid profile, glucose tolerance and lower levels of inflammatory markers. Our analysis does not allow corroborating with these findings, but we can highlight the low percentage in the trajectory of obesity, high glucose and triglyceride in both sexes in the group (0-1) that did not practice sports in youth but is currently active compared to the groups (1-0) and (0-0). This result can be explained by the adaptations in the metabolism of lipids and glucose due to the greater energy demand caused by physical activity (25).

The association analysis (Table 3) showed a protective factor for the obesity trajectory and low HDL-c in the group (1-1) who reported having practiced sports in their youth and are currently active. The literature describes that regular physical activity can increase HDL-c. This modulation is explained by increasing its synthesis and decreasing its degradation in the liver (26). Physical activity can also attenuate the decrease in HDL-c that occurs with advancing age (27).

The reduction of body fat during childhood due to participation in sports activities may have generated a stability between body fat and cardiovascular risk, which may explain the prolonged protective effect of physical activity (28). In contrast, structural theoretical models have pointed out that the protective effect of physical activity from childhood to adolescence depends on the presence of other variables such as current physical activity, obesity and cardiorespiratory fitness (29). Others studies that analyzed the trajectory of physical activity practice pointed out that the interruption of this behavior can cause damage to health, similar to those who remain inactive (30, 31).

Therefore, that maintaining physical activity is important at all stages of life. Aggio et al. (10) and Elhakeem et al. (24) using the trajectories models observed that physical activity performed in older ages is largely dictated by physical activity performed in middle age. Thus, to encouraging sports in childhood and adolescence is essential to develop programs to encourage the practice of physical activity in adults, since being sedentary is always a modifiable risk factor.

Although physical activity incentive programs for workers are challenging due to irregular schedules, early adoption and long-term maintenance of physical activity, can provide greater benefits for the prevention of cardiovascular diseases. Studies describe that an active lifestyle and prevention of chronic diseases can impact the workforce, with less turnover, reduced absences due to medical certificates and reduced early retirements (32, 33).

The evaluation of physical activity in an indirect way and the retrospective analysis of the practice of sports in childhood and adolescence are limitations of the study. Although it is a methodology used in retrospective studies. Memory may be an important bias factor in the analysis of results. Physical activity could be analyzed directly in the three moments and structured in prospective studies. However, the current study obtained the registration of all data on the assessment of cardiovascular risk factors at the three moments, allowing the classification of workers by the trajectory model without the need for data imputation being one of the strengths of this study.

One of the great strengths of the research is that it has a Longitudinal characteristic, and a negative point is that the sample size is relatively small and also that the study is retroactive with characteristic recall biases. Another strength to be considered is the geographic area of the study, which is interesting. The results partially support the conclusions because the Authors did not consider additional protective factors (i.e., diet or sleep or more in general lifestyle). The limitation of not considering risk factors for smoking and alcohol consumption is assumed. These issues should be addressed in future studies that address the same topic. These issues should be addressed in future studies addressing the same theme.

## Conclusions

The study reveals that physical activity patterns and their association with cardiovascular risk factors, within which it was demonstrated that the practice of sports in youth and current physical activity is a protective factor against the trajectory of obesity and low HDL-C.

## Data Availability Statement

The original contributions presented in the study are included in the article/supplementary material, further inquiries can be directed to the corresponding author/s.

## Ethics Statement

The studies involving human participants were reviewed and approved by Research Ethics Committee of the State University of Maringá under opinion number 1,766.685. The patients/participants provided their written informed consent to participate in this study.

## Author Contributions

RM, WR, CC, and AS designed the study. LR, WR, JS, AS, AI, WL, CC, and RM participated in the design and coordination and writing of the manuscript. All authors read and approved the final version of the manuscript, and agreed with the authors order of presentation. All authors contributed to the article and approved the submitted version.

## Funding

The present work was carried out with the support of the State University of Maringá, through the public notice in support of scientific publication, which partially financed this publication; the Coordination for the Improvement of Higher Education Personnel - Brazil (CAPES) - Financing Code 001. Thanks to the State University of Northern Paraná - UENP/PROPG/EDITORA UENP, as a source of partial support for the work. The authors would like to express their rights to the Coordination for the Improvement of Higher Education Personnel (CAPES) and the Associate Graduate Program in Physical Education UEM / UEL (PPGEF) by paying the publication fee.

## Conflict of Interest

The authors declare that the research was conducted in the absence of any commercial or financial relationships that could be construed as a potential conflict of interest.

## Publisher's Note

All claims expressed in this article are solely those of the authors and do not necessarily represent those of their affiliated organizations, or those of the publisher, the editors and the reviewers. Any product that may be evaluated in this article, or claim that may be made by its manufacturer, is not guaranteed or endorsed by the publisher.
